# Basil seedling production environment influences subsequent yield and flavor compound concentration during greenhouse production

**DOI:** 10.1371/journal.pone.0273562

**Published:** 2022-08-25

**Authors:** Kellie J. Walters, Roberto G. Lopez

**Affiliations:** 1 Department of Plant Sciences, University of Tennessee, Knoxville, TN, United States of America; 2 Department of Horticulture, Michigan State University, East Lansing, MI, United States of America; Universite d’Orleans, FRANCE

## Abstract

Radiation intensity and carbon dioxide (CO_2_) concentration can be precisely controlled to manipulate plant yield and quality. Due to increased plant densities during seedling production, fewer inputs per plant are required, creating the potential to increase production efficiency. Therefore, the objectives of this research were to: 1) quantify the extent radiation intensity and CO_2_ concentration under sole-source lighting influence morphology and yield of sweet basil (*Ocimum basilicum*) seedlings, and 2) determine if differences in morphology, yield, and volatile organic compound (VOC) concentration persist after transplant in a common environment. Sweet basil ‘Nufar’ seedlings were grown in growth chambers with target CO_2_ concentrations of 500 or 1,000 μmol·mol^‒1^ under light-emitting diodes (LEDs) providing target photosynthetic photon flux densities (*PPFD*) of 100, 200, 400, or 600 μmol·m^‒2^·s^‒1^ for 16 h per day. After two weeks, seedlings were transplanted into a common greenhouse environment and grown until harvest. At transplant and three weeks after transplant (harvest), growth and developmental differences were quantified along with key terpenoid and phenylpropanoid concentrations at harvest. Radiation intensity and CO_2_ interacted influencing many aspects of plant morphology, though CO_2_ concentration effects were less pronounced than those of radiation intensity. As radiation intensity during seedling production increased from 100 to 600 μmol·m^‒2^·s^‒1^, basil seedlings were 38% taller, had a 713% larger leaf area, and had 65% thicker stems; at harvest, plants were 24% taller, had 56% more branches, 28% more nodes, 22% thicker stems, and weighed 80% more when fresh and dry. Additionally, after growing in a common environment for three weeks, eugenol concentration was greater in plants grown under a *PPFD* of 600 μmol·m^‒2^·s^‒1^ as seedlings compared to lower intensities. Therefore, increasing radiation intensity during seedling production under sole-source lighting can carry over to increase subsequent yield and eugenol concentration during finished production.

## Introduction

Currently, the United States (U.S.) demand for culinary herbs exceeds domestic production, even with controlled environment (CE) production area increasing by 134% and the number of operations increasing by 62% from 2009 to 2014 [1, 2]. Although greenhouses are an example of CEs, it is often difficult to maintain consistent temperatures, radiation levels, and carbon dioxide (CO_2_) concentrations throughout the year [3]. This in turn makes consistent year-round production of food crops challenging. Indoor plant factories and vertical farms can be more precisely controlled, especially for difficult to grow (i.e., tissue-culture transplants) and high-value young plants, to improve uniformity and quality while reducing production time and losses. However, the energy cost of greenhouse heating, lighting, and fans, etc. is often less than that incurred with sole-source lighting, heating, ventilation, and air conditioning used in indoor production systems [4]. Recent advances and increased efficacy of light-emitting diode (LED) fixtures have made sole-source lighting and indoor production more feasible for certain types of production [5].

With more expensive capital and operating costs for indoor production, production of short duration and high-density crops is potentially more profitable [6]. For example, a 200-cell tray of culinary herb seedlings can be produced in two weeks in the same CE area that three to 20 plants can be grown and harvested in three weeks. If the higher operating and capital cost can be spread across a larger number of plants with a shorter production duration, the cost per plant is less. Under indoor sole-source lighting, radiation intensity, temperature, and CO_2_ concentration can be precisely controlled to improve growth, development, and volatile organic compound (VOC) concentrations. However, there is currently limited information on physiological and biochemical responses of culinary herbs to varying radiation intensities and CO_2_ concentrations under sole-source lighting.

With the high input costs during indoor production, maximizing photosynthesis and biomass production are often correlated and integral for production optimization [4]. In C_3_ plants such as basil, biomass production is largely determined by radiation intensity and CO_2_ concentration, as these two parameters influence carbon accumulation through photosynthesis and photorespiration [7]. Research towards optimizing CO_2_ and radiation intensity or daily light integral (DLI) for CE basil production has been ongoing. Many researchers have quantified increases in fresh mass as light intensity increases, but most did not include saturating light intensities [8–10]. Those who do quantify saturating intensities report varied optimal intensities for biomass production including 250 μmol·m^–2^·s^–1^ (14.4 mol·m^–2^·d^–1^ DLI) [11], 500 μmol·m^–2^·s^–1^ (28.8 mol·m^–2^·d^–1^ DLI) [12], and 600 μmol·m^–2^·s^–1^ (38.9 mol·m^–2^·d^–1^ DLI) [13]. Additionally, increasing CO_2_ concentrations during basil production from 360 to 620 μmol·mol^–1^ and from 420 to 720 μmol·mol^–1^ increased fresh mass [14, 15], but the interaction of CO_2_ concentration and radiation intensity in basil has not been thoroughly explored.

In addition to biomass production, crop quality, especially improved flavor, is an integral goal of crop production. CE growers have indicated a need for research on adjusting the growing environment to improve crop flavor [16, 17]. Many VOCs contribute to basil flavor, including phenylpropanoids and terpenoids. Eugenol is a phenylpropanoid that contributes a clove-like flavor and aroma, while methyl chavicol (estragole) is more anise-like [18]. The terpenoid linalool can be described as floral or spicy [19] or reminiscent of the cereal “Fruit Loops^®^”. 1,8 Cineole, another terpenoid, contributes an aroma and flavor analogous to eucalyptus (*Eucalyptus globulus*) [20].

Daily light integral and CO_2_ concentration influence secondary metabolite concentrations, including VOCs. Dou et al. [8] determined that increasing DLI from 9.3 to 17.8 mol·m^–2^·d^–1^ not only increased basil fresh mass, net photosynthesis, and leaf area and thickness, but also increased anthocyanin, phenolic, and flavonoid concentrations. Chang et al. [21] reported that as the DLI delivered for two weeks to young seedlings increased from 5.3 to 24.9 mol·m^–2^·d^–1^, total VOC content of basil increased. In particular, the relative content of eugenol and linalool increased ~300% and ~400%, respectively, while methyl eugenol relative content decreased by ~80%. Carbon dioxide can also influence secondary metabolite production. For example, linalool, a compound of interest in basil, is also present in strawberry (*Fragaria ananassa*) [22]. An increase in CO_2_ concentration from ~350 to ~950 μmol·mol^–1^ increased linalool concentration during strawberry production [22]. By investigating the individual and combined influences of DLI and CO_2_ on VOC content and concentration, CE growers can work toward optimizing growing conditions that increase plant quality (VOC content and concentration) and yield (fresh mass).

Although technological advances have made indoor plant production more economically feasible, a better understanding of how to leverage environmental controls to improve crop productivity, quality, and energy efficiency is needed [23]. While researchers have been mainly focused on investigating the influence of environmental variables on finished-stage crops, the potential to improve high-density young plant production creates an opportunity to spread potentially greater input costs over a larger number of plants. Therefore, the objectives of this research were to: 1) quantify the extent radiation intensity and CO_2_ concentration during seedling production influence yield, 2) determine if physiological and morphological differences remain present after transplant in a greenhouse, and 3) determine if differences in VOC concentration due to radiation intensity at the seedling stage remain present through harvest in a common greenhouse environment. Our hypotheses were that 1) growth would increase as radiation intensity and CO_2_ increased, 2) there would be a positive interactive effect between CO_2_ concentration and radiation intensity, where the effects of elevated CO_2_ concentration would be more pronounced when the radiation intensity was higher, and 3) increased VOC concentrations at transplant would not persist through finishing in a common environment due to dilution during rapid growth.

## Materials and methods

### Seedling production

Sweet basil ‘Nufar’ (Johnny’s Selected Seeds, Fairfield, ME) was selected based on disease resistance and comparatively high yield results from Walters and Currey [24]. Seeds were sown two per cell in stone wool cubes (2.5 × 2.5 × 4 cm, AO plug; Grodan, Roermond, Netherlands) and 200-cell flats were placed in one of two walk-in growth chambers (Hotpack environmental room UWP 2614–3; SP Scientific, Warminster, PA) on 7 Aug. 2017, 10 Nov. 2017, and 22 Jan. 2018. Seeds and seedlings were grown and the environmental conditions were controlled and monitored as reported in Walters et al. [25].

**Fig 1:**
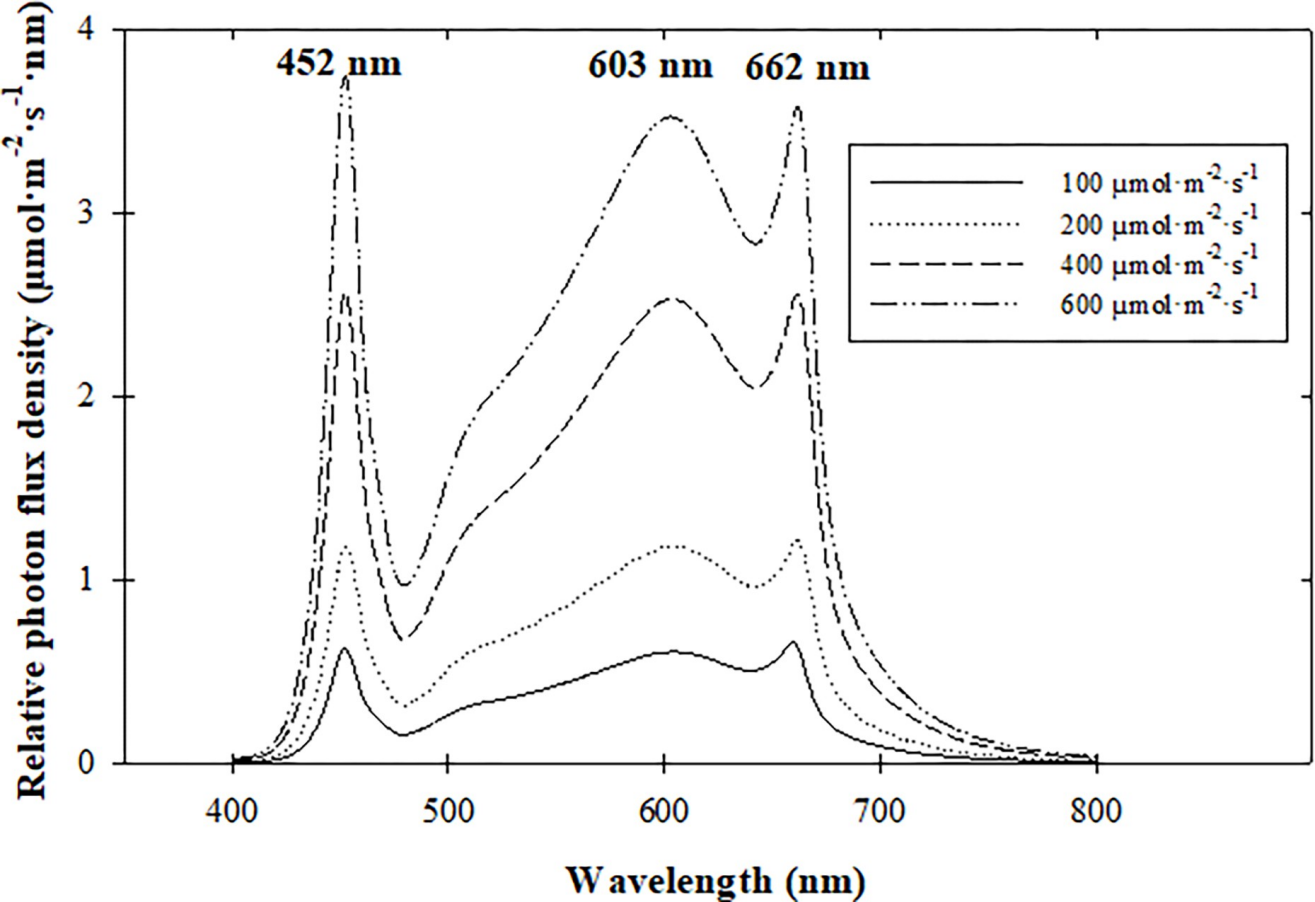
Spectral quality of broad-spectrum light-emitting diode (LED) fixtures providing 20:40:40 blue:green:red radiation ratios (%), a red:far-red ratio of 13:1, and target radiation intensities of 100, 200, 400, or 600 μmol·m^–2^·s^–1^.

Light-emitting diodes (LEDs) provided 20:40:40 blue:green:red radiation ratios (%), a red:far-red ratio of 13:1, and target radiation intensities of 100, 200, 400, or 600 μmol·m^‒2^·s^‒1^ photosynthetic photon flux density (*PPFD*) for a 16-h photoperiod to create daily light integrals (DLIs) of 6, 12, 23, or 35 mol·m^‒2^·d^‒1^. Fixture density and hanging height were adjusted to achieve target radiation intensities. Radiation intensity and spectrum were measured at four corners and in the center of the seedling flat with a spectroradiometer (PS-200; StellarNet, Inc., Tampa, FL) to quantify the intensities and spectrum across the growing area (Fig 1, Table 1). Target carbon dioxide concentrations of 500 and 1000 μmol·mol^–1^ were maintained by injecting compressed CO_2_ to increase concentrations and scrub CO_2_ using soda lime scrubber (Environmental Growth Chambers) to decrease concentrations. Concentrations were measured with a CO_2_ sensor (GM86P; Vaisala, Helsinki, Finland) and logged by a C6 Controller (Environmental Growth Chambers) every 5 s (Table 1).

**Table 1:** The date of sweet basil ‘Nufar’ (*Ocimum basilicum*) seed sowing, target and actual CO_2_ concentration (± sd), target and actual radiation intensity (± sd), and average daily air, canopy, and substrate temperature (± sd) during the seedling growth stage (2 weeks).

Rep & start date	CO_2_ (μmol·mol^–1^)		Radiation intensity (μmol·m^‒2^·s^‒1^)		Temperature (°C)		
	Target	Actual	Target	Actual	Air	Canopy	Substrate
1	500	503 ± 14	100	94 ± 4	23.0 ± 0.6	25.1 ± 0.6	23.1 ± 0.4
7 Aug. 2017			200	200 ± 2		24.4 ± 0.6	23.0 ± 0.5
			400	413 ± 6		26.8 ± 1.4	24.1 ± 1.2
			600	614 ± 13		27.7 ± 1.8	25.0 ± 1.6
	1000	991 ± 19	100	102 ± 1	23.0 ± 0.1	24.2 ± 0.7	23.7 ± 0.8
			200	193 ± 4		26.3 ± 1.0	-^z^
			400	423 ± 25		27.1 ± 1.4	24.2 ± 1.2
			600	589 ± 13		28.9 ± 1.7	24.7 ± 1.4
2	500	504 ± 11	100	94 ± 4	22.9 ± 1.8	24.5 ± 0.9	22.2 ± 1.0
10 Nov. 2017			200	188 ± 2		24.0 ± 1.1	22.0 ± 0.9
			400	432 ± 1		26.9 ± 1.5	23.7 ± 1.4
			600	615 ± 2		27.7 ± 2.1	24.6 ± 1.9
	1000	1016 ± 14	100	102 ± 0	23.0 ± 0.0	24.4 ± 0.9	21.9 ± 0.7
			200	191 ± 0		26.4 ± 1.0	-
			400	429 ± 1		27.4 ± 1.7	24.1 ± 1.3
			600	577 ± 2		28.7 ± 1.7	25.2 ± 1.9
3	500	506 ± 23	100	88 ± 2	23.0 ± 1.4	24.8 ± 0.9	22.5 ± 0.8
22 Jan. 2018			200	191 ± 3		24.1 ± 0.8	22.7 ± 1.0
			400	394 ± 7		26.9 ± 1.5	23.5 ± 1.4
			600	589 ± 11		27.9 ± 2.2	24.6 ± 1.7
	1000	1017 ± 29	100	99 ± 2	23.0 ± 0.4	24.2 ± 0.5	21.7 ± 0.6
			200	184 ± 3		26.5 ± 0.9	-
			400	384 ± 6		27.4 ± 1.6	24.3 ± 1.5
			600	555 ± 9		29.3 ± 2.0	25.3 ± 1.9

^z^ Data not collected.

### Finished plant production

Two weeks after sowing, 17 seedlings were transplanted into 0.9-m-wide by 1.8-m-long deep-flow hydroponic systems (Active aqua premium high-rise flood table; Hydrofarm, Petaluma, CA) in a glass-glazed greenhouse. Baskets holding the seedlings were placed in 4-cm-diameter holes, 20-cm apart, in 4-cm thick extruded polystyrene foam floating on the nutrient solution. The nutrient solution consisted of reverse osmosis water supplemented with 12N-1.8P-13.4K water-soluble fertilizer (RO Hydro FeED; JR Peters, Inc.) and MgSO_4_ providing twice the concentrations reported during seedling production. Electrical conductivity (EC) and pH were measured (HI991301 Portable Waterproof pH/EC/TDS Meter; Hanna Instruments, Woonsocket, RI) and adjusted to 1.56 mS·cm^–1^ and 6.0, respectively, by adding fertilizer, reverse osmosis water, potassium bicarbonate, or sulfuric acid. Air pumps (Active aqua 70 L·min^–1^ commercial air pump; Hydrofarm) and air stones (Active aqua air stone round 4”x1”; Hydrofarm) were used to increase dissolved oxygen concentrations.

The 16-h (0600 to 2200 hr) photoperiod consisted of natural photoperiods (lat. 43° N) and day-extension lighting from high-pressure sodium (HPS) lamps providing a supplemental *PPFD* of ~75 μmol·m^–2^·s^–1^ to achieve target DLIs of 13 to 17 mol·m^–2^·d^–1^. The target average daily temperature was a constant 23°C. Exhaust fans, evaporative-pad cooling, radiant steam heating, and supplemental lighting was controlled by an environmental control system (Integro 725; Priva North America, Vineland Station, ON, Canada). Shielded and aspirated 0.13-mm type E thermocouples (Omega Engineering) measured air temperature, infrared thermocouples (OS36-01-T-80F; Omega Engineering) measured leaf temperature, and quantum sensors (LI-190R Quantum Sensor; LI-COR Biosciences) placed at canopy height recorded *PPFD*. Every 15 s, a CR-1000 datalogger (Campbell Scientific) collected environmental data and hourly means were recorded (Table 2).

**Table 2:** Actual average daily air and canopy temperature and daily light integral (DLI) (mean ± sd) during post-transplant greenhouse production (3 weeks of sweet basil ‘Nufar’ (*Ocimum basilicum*).

	Temperature (°C)		DLI
Rep.	Air	Canopy	mol·m^‒2^·d^‒1^
1	21.6 ± 2.1	26.1 ± 3.5	14.1 ± 1.8
2	22.6 ± 1.3	22.6 ± 1.9	12.9 ± 3.2
3	23.1 ± 1.1	22.5 ± 2.4	17.4 ± 4.5

### Growth, development, and VOC data collection and analysis

At transplant and three weeks after transplant (harvest), height from the substrate surface to the meristem, leaf area of two (seedling) or four (harvest) most recently fully expanded leaves (measured with LI-300; LI-COR Biosciences), stem diameter (harvest reps 2 and 3) at the base, and shoot fresh mass were recorded. Additionally, the number of branches >2.5 cm and node number (rep 2 and 3) were recorded at harvest. Tissue was placed in a forced-air oven maintained at 75°C for at least 3 d, weighed, and dry mass was recorded. Three weeks after transplant, the two most recent, fully mature leaves of five plants from each treatment were detached, frozen, and stored at -20°C until gas chromatography mass spectrometry (GCMS) analysis as reported in Walters et al. [25] from a method derived from [26] Schilmiller et al. Tissue was ground in liquid nitrogen, and compounds were extracted with methyl *tert*-butyl ether (MTBE) with a tetradecane internal standard. Samples were analyzed using an Agilent 7890A GC and single quadrupole MS with 5975C inert XL MS detector (Agilent, Santa Clara, CA). Compound concentrations were normalized to the sample internal standard and leaf dry weight, then quantified using the standard calibration curves of 1,8 cineole, eugenol, linalool, and methyl chavicol with a tetradecane internal standard (Millipore Sigma; St. Louis, MO).

### Statistical design and analysis

The seedling portion of this experiment was organized the same as Walters et al. [25], a split-plot design with two CO_2_ concentrations (two growth chambers) as the main factor and four radiation intensities as the sub factor. Finished greenhouse production was organized in a randomized complete block design with seedlings from the growth chamber blocked by treatment. The experiment was completed thrice over time for growth and development analysis (n = 30), and twice in time for GCMS analysis (reps 2 and 3; n = 20). Analysis of variance and t-tests were performed using JMP (version 12.0.1, SAS Institute Inc., Cary, NC). When interactions (CO_2_ concentration × radiation intensity) were not significant, data were pooled (n = 60). Linear and quadratic regression analyses were conducted using Sigma Plot (version 11.0, Systat Software Inc., San Jose, CA).

## Results

### Seedlings

**Fig 2:**
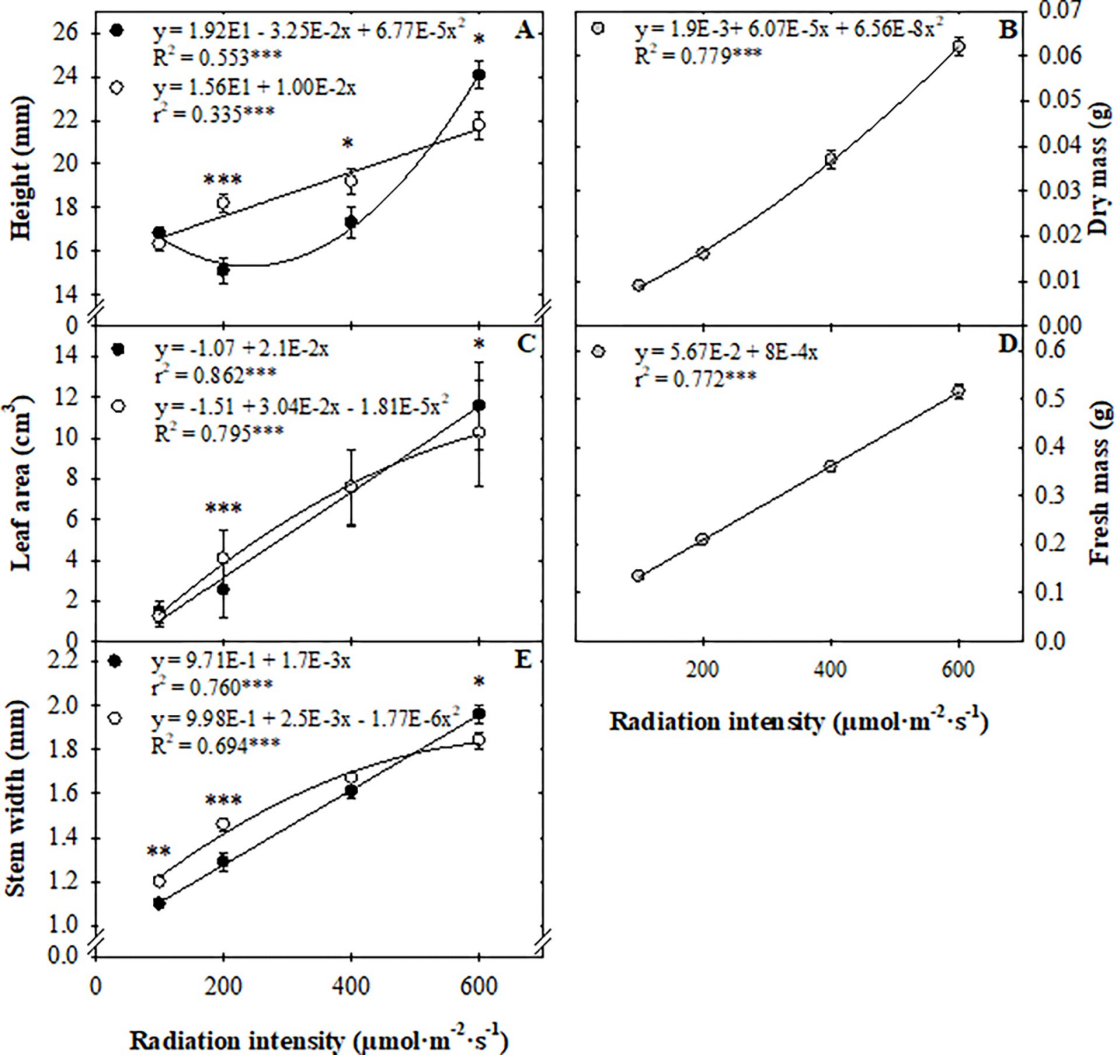
Radiation intensity (100, 200, 400, or 600 μmol·m^–2^·s^–1^) for a 16-h photoperiod to create daily light integrals of (6, 12, 23, or 35 mol·m^‒2^·d^‒1^) and CO_2_ concentration (, 500 μmol·mol^–1^; 1,000 μmol·mol^–1^; pooled) effects on sweet basil ‘Nufar’ (*Ocimum basilicum*) seedling height (A), dry mass (B), leaf area (C), fresh mass (D), and stem width (E) two weeks after sowing. Lines represent linear or quadratic regressions. Symbols (means ± se) represent measured data (and, n = 30; n = 60). *, **, and *** indicate significant at *P* ≤ 0.05, 0.01, or 0.001, respectively.

Radiation intensity but not CO_2_ concentration influenced fresh and dry mass (Fig 2B and 2D). Fresh mass increased linearly from 0.134 to 0.515 g (284%) and dry mass increased quadratically from 0.009 to 0.062 g (589%) as radiation intensity increased from 100 to 600 μmol·m^–2^·s^–1^.

Chamber CO_2_ concentration and radiation intensity interacted to affect height, leaf area, and stem width. As radiation intensity increased, height, leaf area, and stem width generally increased (Fig 2A, 2C and 2E). As radiation intensity increased from 100 to 600 μmol·m^–2^·s^–1^, basil seedlings were 38% (0.6 cm) taller, had a 713% (9.6 cm^3^) larger leaf area, and 65% (0.8 mm) thicker stems. Seedlings grown under 100 μmol·m^–2^·s^–1^ were a similar height (1.6 cm) when grown at 500 or 1,000 μmol·mol^–1^ CO_2_. Plants grown under 200 μmol·m^–2^·s^–1^ were 0.3 cm taller when grown at 1,000 than 500 μmol·mol^–1^ CO_2_, and plants grown under 600 μmol·m^–2^·s^–1^ were 0.2 cm shorter when grown at 1,000 than 500 μmol·mol^–1^ CO_2_ (Fig 2A). Similarly, when grown under lower radiation intensities (100 or 200 μmol·m^–2^·s^–1^), stem width was 0.1 mm greater with a CO_2_ concentration of 1,000 than 500 μmol·mol^–1^. However, when plants were grown under higher intensities (600 μmol·m^–2^·s^–1^), stem width was 0.1 mm greater at 500 μmol·mol^–1^ compared to 1,000 μmol·mol^–1^ CO_2_ (Fig 2E). Under a radiation intensity of 200 μmol·m^–2^·s^–1^, leaf area of seedlings was 3.1 cm^3^ larger at 1,000 μmol·mol^–1^ than at 500 μmol·mol^–1^ CO_2_, while the leaf area was 2.3 cm^3^ smaller under 600 μmol·m^–2^·s^–1^ (Fig 2C).

### Harvest

**Fig 3:**
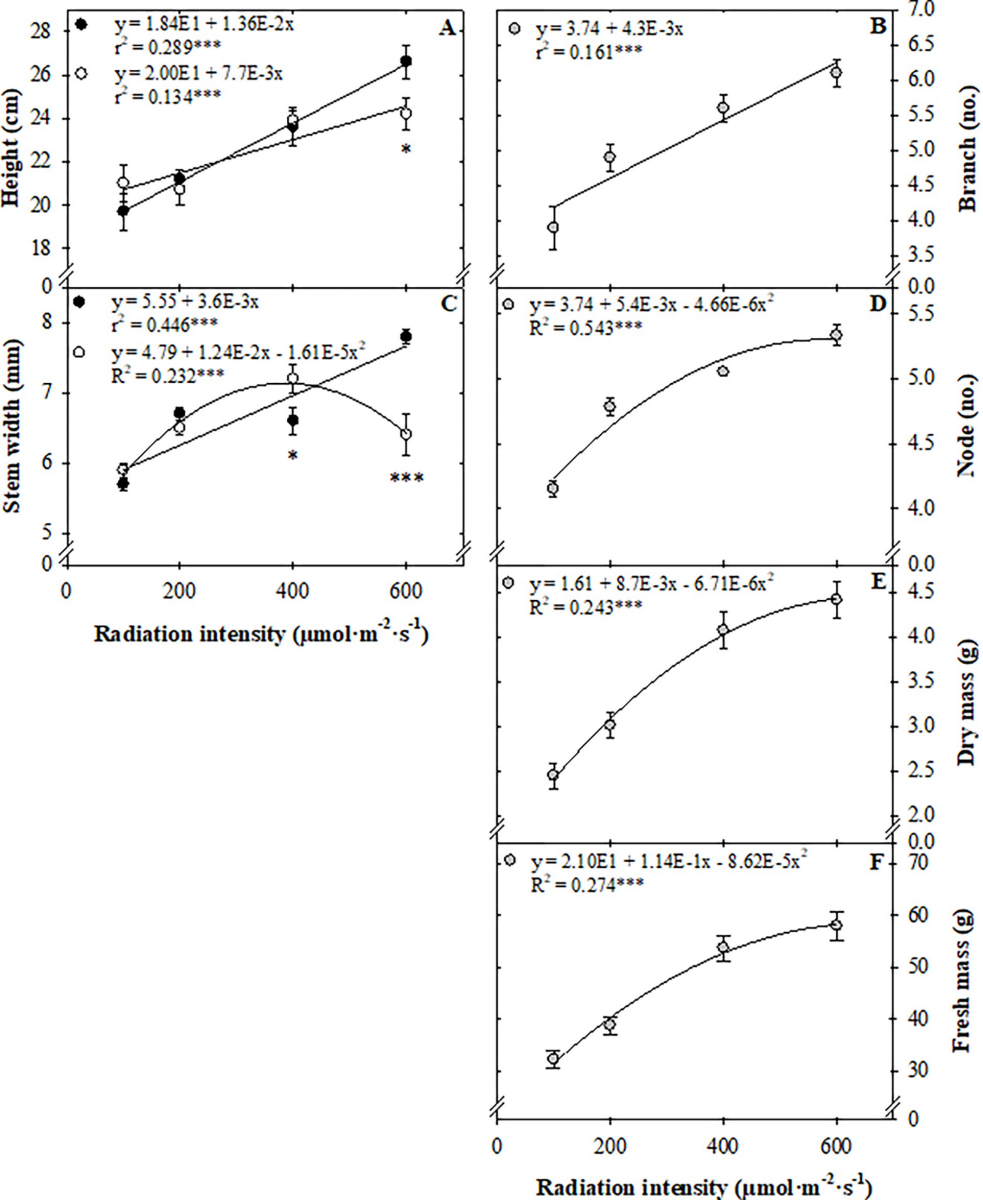
Radiation intensity (100, 200, 400, or 600 μmol·m^–2^·s^–1^) for a 16-h photoperiod to create daily light integrals of (6, 12, 23, or 35 mol·m^‒2^·d^‒1^) and CO_2_ concentration (, 500 μmol·mol^–1^; 1,000 μmol·mol^–1^; pooled) administered during seedling production, two weeks after sowing. The figures depict seedling treatment effects on sweet basil ‘Nufar’ (*Ocimum basilicum*) height (A), branch number (B), stem width (C), node number (D), dry mass (E), and fresh mass (F) three weeks after transplant into a common enviornment. Lines represent linear or quadratic regressions. Symbols (means ±se) represent measured data (and, n = 30; n = 60). * and *** indicate significant at *P* ≤ 0.05 or 0.001, respectively.

In general, radiation intensity during the seedling stage influenced height, branch and node number, stem width, and fresh and dry mass at harvest, three weeks after transplant (Fig 3A–3F). Plants grown under a radiation intensity of 600 μmol·m^–2^·s^–1^ as seedlings, were 5 cm taller (24% increase), and had 2 more branches (56% increase), 1 more node (28% increase), 1.3- mm thicker stems (22% increase), 25 g more fresh mass (80% increase), and 2 g more dry mass (80% increase) at harvest compared to plants grown under 100 μmol·m^–2^·s^–1^ as seedlings. Leaf area of the four leaves measured at harvest was not affected by radiation intensity or CO_2_ concentration during the seedling stage (data available in GitHub).

Similar to the seedling stage, radiation intensity and CO_2_ concentration interacted to affect height and stem width at harvest (Fig 3A and 3C). Plants grown under 100 or 200 μmol·m^–2^·s^–1^ during the seedling stage had similar height and stem width at harvest regardless of CO_2_ concentration. Basil grown under a *PPFD* of 600 μmol·m^–2^·s^–1^ as seedlings were 2.4 cm (10%) taller and had 1.4 mm (22%) thicker stems when grown at 500 μmol·mol^–1^ compared to 1,000 μmol·mol^–1^ CO_2_ (Fig 3A and 3C).

### Finished volatile organic compound concentrations

**Fig 4:**
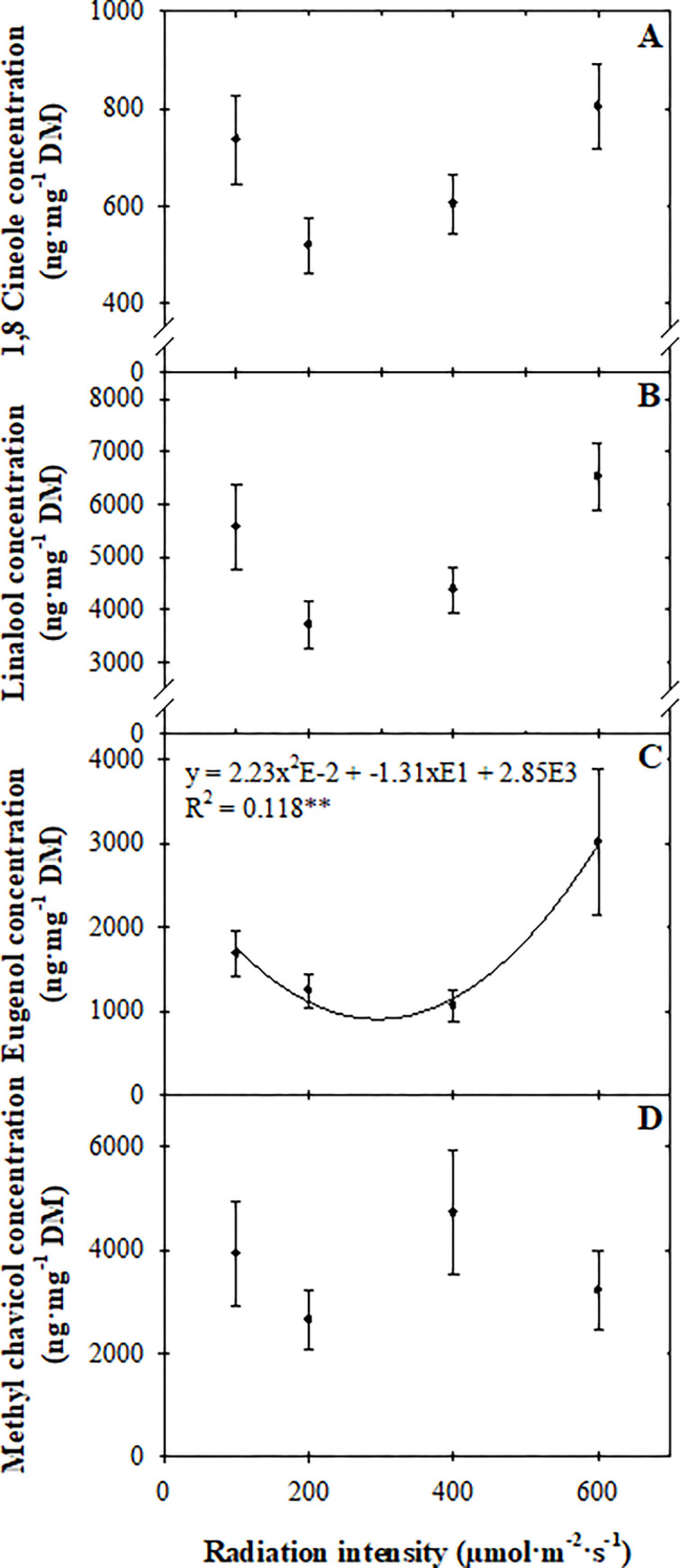
Concentrations [ng·mg^‒1^ dry mass (DM)] of 1,8 cineole (A), linalool (B), eugenol (C), and methyl chavicol (D) of sweet basil ‘Nufar’ (*Ocimum basilicum*) seedlings grown under radiation intensity (100, 200, 400, or 600 μmol·m^–2^·s^–1^) for a 16-h photoperiod to create daily light integrals of (6, 12, 23, or 35 mol·m^‒2^·d^‒1^) for two weeks and then transplanted in a common greenhouse environment and grown for three weeks. Each symbol represents the mean of 20 plants ± se. Lines represent linear or quadratic regression. ** indicates significant at *P* ≤ 0.01.

After seedlings were transplanted into a common greenhouse environment and grown for three weeks, there was no difference in linalool, 1,8 cineole, or methyl chavicol concentrations due to radiation intensity or CO_2_ concentration provided during the seedling stage (Fig 4A, 4B and 4D). However, as the radiation intensity during seedling production increased, an overall quadratic increase in eugenol concentration persisted through finishing (Fig 4C). There were minimal differences in eugenol concentration among plants grown under a *PPFD* of 100, 200, or 400 μmol·m^‒2^·s^‒1^ during the seedling stage, three weeks after transplant. Increasing the radiation intensity to 600 μmol·m^‒2^·s^‒1^ during the seedling stage increased eugenol concentration 44% to 183% (1,327 to 1,946 ng·mg^‒1^ dry mass) compared to the lower radiation intensity treatments. There was no effect of CO_2_ concentration on VOCs.

## Discussion

### Increased radiation intensity increased growth and morphological attributes

Increasing radiation intensity or DLI up to a saturating value increases biomass production. Walters and Currey [10] reported that sweet basil ‘Nufar’ fresh mass increased 144% as DLI increased from 7 to 15 mol·m^‒2^·d^‒1^. Increasing DLI from 9.3 to 17.8 mol·m^‒2^·d^‒1^ during sweet basil ‘Improved Genovese Compact’ production increased fresh mass 78% [8], increasing DLI from 2 to 20 mol·m^‒2^·d^‒1^ increased sweet basil ‘Nufar’ fresh mass 24-fold [9], and increasing the DLI from 5.8 to 14.4 mol·m^‒2^·d^‒1^ increased ‘Genovese’ fresh mass 3-fold [11]. However, none of these studies increased DLI above 20 mol·m^‒2^·d^‒1^. The goal of Beaman et al. [12] was to determine the radiation intensity that led to the highest sweet basil biomass production. Sweet basil ‘Nufar’ shoot fresh mass increased 39% as radiation intensity increased from a *PPFD* of 300 to 500 μmol·m^‒2^·s^‒1^ (DLIs of 17.3 to 28.8 mol·m^‒2^·d^‒1^), while fresh mass was similar among plants grown under 500 and 600 μmol·m^‒2^·s^‒1^ (28.8 and 34.6 mol·m^‒2^·d^‒1^). Though these previously mentioned studies were conducted at harvest on plants in the finished stage of production, our study with seedlings concurs; increasing radiation intensity from 100 to 600 μmol·m^‒2^·s^‒1^ (DLI of 5.8 to 34.6 mol·m^‒2^·d^‒1^) increased seedling fresh mass by 284% (Fig 2D). Our results confirm that regardless of production stage, radiation intensity during sweet basil production can be increased up to 600 μmol·m^‒2^·s^‒1^ (34.6 mol·m^‒2^·d^‒1^) to increase fresh mass.

### CO_2_ concentration did not influence mass

Contrary to our hypothesis, CO_2_ concentration did not influence fresh or dry mass at transplant or harvest (Figs 2B and 2D, 3E and 3F). While most research illustrates increased CO_2_ concentrations can increase biomass, there are a few reasons plants may not respond to elevated CO_2_ concentrations. Plants can become acclimated to elevated CO_2_ concentrations, with prolonged exposure becoming inhibitory to photosynthesis; however, the extent and presence of acclimation or negative effects are species- and potentially, production stage-dependent [27, 29]. For example, Sage et al. [27] reported that long-term elevated CO_2_ (900 to 1000 μmol·mol^–1^) negatively affected the photosynthetic rate of C_3_ plants such as kidney bean ‘Linden’ (*Phaseolus vulgaris*), eggplant (*Solanum melongena*), and cabbage (*Brassica oleracea*), but increased the photosynthetic rate of C_3_ plant lambsquarters (*Chenopodium album*). While the main benefit of elevated CO_2_ is the favoring of carboxylation activity over oxygenation activity of ribulose-1,5-bisphosphate carboxylase/oxygenase (Rubisco), initial increased photosynthetic rates can cause excess carbohydrate production resulting in feedback inhibition, thus a reduction in photosynthesis [27–29]. Additionally, in some species such as cabbage, lambsquarters, petunia (*Petunia ×hybrida*), elevated CO_2_ decreased Rubisco content and/or activity [27, 29], induced stomatal closure, and/or decreased stomatal density [27, 29, 30].

The lack of CO_2_ effect on biomass could also be due to the concentration being near or above the CO_2_ saturation point. Previous research determined that increasing CO_2_ concentrations from 360 to 620 μmol·mol^–1^ increased ~4-week old basil (cultivar not reported) fresh mass 40% when grown under a radiation intensity of 150 μmol·m^‒2^·s^‒1^ (8.6 mol·m^‒2^·d^‒1^) [15]. We hypothesize that the increased mass with increased CO_2_ concentration reported by Al Jaouni et al. [15] was due to CO_2_ concentrations being below the CO_2_ saturation point. Park et al. [31] determined the CO_2_ saturation point of basil was 729 μmol·m^‒2^·s^‒1^ when sweet basil (cultivar not reported) was acclimated to 20°C, 400 μmol·mol^–1^ CO_2_, and a radiation intensity of 150 μmol·m^‒2^·s^‒1^ (9.7 mol·m^‒2^·d^‒1^). Additionally, as CO_2_ concentration approaches the saturation point, increases in mass are attenuated. Since we utilized 500 and 1,000 μmol·mol^–1^ CO_2_, 500 μmol·mol^–1^ may have been too similar to the CO_2_ saturation point and 1,000 μmol·mol^–1^ may have been above the saturation point, resulting in no discernable difference in mass. If we had maintained CO_2_ concentrations below 500 μmol·mol^–1^, differences may have occurred; however, this hypothesis would have to be tested.

Another contributing factor could be that CO_2_ utilization may be limited by environmental factors including temperature. As temperature increases, Rubisco has a higher affinity for oxygen; therefore, the positive influence of elevated CO_2_ on photosynthesis increases as temperature increases from approximately 20 to 35°C, though the effect is species-dependent [32]. In this study, basil was grown at ~23°C. If temperatures had been higher and closer to the optimal temperature for sweet basil growth and development of 32 to 35°C [33], the elevated CO_2_ concentration may have been more likely to have had an effect on fresh mass.

### CO_2_ concentration influenced morphology

The increased height, leaf area, and stem width due to lower radiation intensities (100 to 400 μmol·m^‒2^·s^‒1^; Fig 2A, 2C and 2E) with elevated CO_2_ concentration were likely due to differing biomass partitioning, since neither fresh nor dry mass were affected. In wheat ‘WW15’ (*Triticum aestivum*), leaf area index increased at elevated CO_2_ concentrations under lower radiation conditions [34]. However, there are conflicting reports on the effect of elevated CO_2_ on leaf area. In separate experiments under different environmental conditions, leaf area of tomato ‘Minibelle’ increased as CO_2_ concentration increased [35], however, tomato ‘Findon Cross’ leaf area was unaffected [36]. The lower height, leaf area, and stem width when basil seedlings were under a radiation intensity of 600 μmol·m^‒2^·s^‒1^ and elevated CO_2_ was counterintuitive (Fig 2A, 2C and 2E). It is well documented that as radiation intensity increases, leaf thickness increases [8]. It could be possible that increased tissue thickness can impact plant responses to elevated CO_2_, however, additional morphological and physiological data is needed to confirm or reject the hypothesis.

### Seedling production conditions influence basil yield and quality at harvest

Increasing radiation intensity from 100 to 600 μmol·m^‒2^·s^‒1^ (5.8 to 34.6 mol·m^‒2^·d^‒1^) increased seedling fresh mass 284% (Fig 2D), and an 80% increase in fresh mass yield persisted through finishing in a common environment (Fig 3C). Radiation intensity during propagation of floriculture crops can have a profound effect on subsequent growth and development. For example, increasing DLI from 4.1 to 14.2 mol·m^‒2^·d^‒1^ during seedling production hastened flowering and reduced shoot dry weight at flower for celosia (*Celosia argentea* var. *plumosa*), impatiens (*Impatiens walleriana*), French marigold (*Tagetes*), and pansy (*Viola*) [37]. However, the reduction in dry mass can be primarily attributed to earlier flowering and thus, a shorter production duration. In the present study, plants were not grown until anthesis, but were harvested at the same time. Therefore, the influence of radiation intensity observed at transplant persisted, but was attenuated. Similar to the floriculture studies, development, including node and branch number, was hastened by increased radiation intensity during propagation in our study.

In addition to increased yield at harvest, and contrary to our hypothesis, the higher eugenol concentrations in seedlings grown under high radiation intensities [25] persisted at harvest (Fig 4C). Walters et al. [25] observed that increasing radiation intensity from 100 to 600 μmol·m^‒2^·s^‒1^ (5.8 to 34.6 mol·m^‒2^·d^‒1^) during basil seedling production increased 1,8 cineole, linalool, and eugenol concentrations. In a study investigating the influence of radiation quality on basil VOCs, researchers suggested that specific light treatments during germination and early seedling development “may install a particular developmental/metabolic pattern that influences potential to produce flavor and aroma compounds later” [38]. Our results suggest that the production or biosynthetic pathway of some compounds may be more sensitive to early environmental conditions than others. From a crop quality perspective, growers have indicated that their customers would pay more for crops with increased flavor [17]. However, consumer sensory panels have determined that when consumed raw and alone, there is an upper limit to consider and consumers do not always prefer basil with a more intense flavor [25]. Therefore, the benefit of an elevated eugenol concentration at harvest is not clear and may be situational.

### Efficiency implications

In this study, we investigated the effect of increased inputs during the seedling stage to increase yield and secondary metabolite accumulation at harvest. By sowing seeds in a 200-cell tray (1,290 cm^2^ per flat, 6.45 cm^2^ per cell), the planting density is 1,550 plants per m^2^. Seedlings were transplanted 20-cm apart with a planting density of 25 plants per m^2^. Therefore, planting density was 62 times greater during propagation than finished (harvest) production. Additionally, in this study, the duration of seedling production was 2/3 that of finishing (two weeks compared to three weeks). Taking both the increased planting density and shorter production duration into account, the increase in lighting cost per plant could be discounted by 93 times during seedling versus finished production.

Therefore, in this case, the cost per plant of increasing the radiation intensity from 100 to 600 μmol·m^‒2^·s^‒1^ during propagation was ~5% that of the cost during finished production. Since the increase in yield at the finishing stage was 80% greater when seedlings were grown under 600 μmol·m^-2^·s^-1^ compared to 100 μmol·m^-2^·s^-1^ (34.6 compared 5.8 mol·m^‒2^·d^‒1^), increasing radiation intensity during propagation increases subsequent yield and eugenol concentration while reducing costs.

## Conclusions

Increasing radiation intensity from 100 to 600 μmol·m^‒2^·s^‒1^ (5.8 to 34.6 mol·m^‒2^·d^‒1^) during basil seedling production is an effective method of improving subsequent yields and increasing eugenol concentration. Although elevated CO_2_ concentrations did not influence fresh or dry mass, future research is needed to determine at what stage of production elevated CO_2_ concentrations could increase basil growth and secondary metabolite concentrations, if any. With these data, environmental controls, especially radiation intensity and CO_2_ concentration, can be better leveraged during young plant production to improve crop productivity, quality, and energy efficiency not only at transplant, but also after finishing in a common environment. Additionally, this research can serve as a basis for scientific advances in dynamic environmental control.
